# A dimeric state for PRC2

**DOI:** 10.1093/nar/gku540

**Published:** 2014-07-03

**Authors:** Chen Davidovich, Karen J. Goodrich, Anne R. Gooding, Thomas R. Cech

**Affiliations:** Department of Chemistry & Biochemistry, Howard Hughes Medical Institute, University of Colorado BioFrontiers Institute, Boulder, CO 80309–0596, USA

## Abstract

Polycomb repressive complex-2 (PRC2) is a histone methyltransferase required for epigenetic silencing during development and cancer. Long non-coding RNAs (lncRNAs) can recruit PRC2 to chromatin. Previous studies identified PRC2 subunits in a complex with the apparent molecular weight of a dimer, which might be accounted for by the incorporation of additional protein subunits or RNA rather than PRC2 dimerization. Here we show that reconstituted human PRC2 is in fact a dimer, using multiple independent approaches including analytical size exclusion chromatography (SEC), SEC combined with multi-angle light scattering and co-immunoprecipitation of differentially tagged subunits. Even though it contains at least two RNA-binding subunits, each PRC2 dimer binds only one RNA molecule. Yet, multiple PRC2 dimers bind a single RNA molecule cooperatively. These observations suggest a model in which the first RNA binding event promotes the recruitment of multiple PRC2 complexes to chromatin, thereby nucleating repression.

## INTRODUCTION

Polycomb repressive complex-2 (PRC2) is required for epigenetic silencing of transcription during embryonic development and cancer (reviewed in (1–3)). It is a histone methyltransferase that mono-, di- and tri-methylates lysine 27 of histone H3 (H3K27me1, H3K27me2 and H3K27me3, respectively), epigenetic marks associated with repressed chromatin. EZH2 is the catalytic subunit of PRC2, and SUZ12 is an essential regulatory subunit (4). EED is a histone-binding subunit that binds H3K27me3-modified histone tails, resulting in increased affinity to nucleosomes and stimulation of the catalytic activity of PRC2 (5,6). Marks for active chromatin, H3K4me3 and H3K36me3, are recognized by RBBP4 together with SUZ12 to promote catalytic inhibition of PRC2 and reduced affinity to nucleosomes (7,8). While PRC2 is commonly studied as a complex of these four subunits (namely, PRC2 4m), AEBP2 is a zinc-finger protein that associates with PRC2 and is suggested to comprise a fifth subunit of PRC2 (4,9) (i.e. PRC2 5m); AEBP2 may have a role in DNA binding (10).

In *Drosophila* PRC2 is recruited to chromatin through polycomb response elements (reviewed in (11)). Despite the discovery of functionally similar elements in vertebrates (12–14), the understanding of PRC2-specific recruiters to chromatin is far from complete. Evidence indicates that long non-coding RNAs (lncRNAs) recruit PRC2 to loci designated for silencing. HOTAIR lncRNA recruits PRC2 *in trans* to the HOXD locus (15,16) and to various other loci (17). RepA ncRNA recruits PRC2 during X-chromosome inactivation (18). Moreover, PRC2 is associated with thousands of RNAs in various cell lines (16,18–22). A two-hairpin motif has been suggested to be enriched in a subclass of non-coding RNAs that associate with PRC2 (19), inspired by a two-hairpin motif that was originally discovered in RepA RNA (18). However, diverse RNAs that do not contain the two-hairpin motif bind PRC2 with similar affinity. Promiscuous binding of PRC2 to RNA was shown *in vitro* (23) and *in vivo* (23,24) and has been proposed to provide a mechanism for maintenance of the repressed chromatin state (23).

Gel filtration fractionation of nuclear extracts from mouse or human cells or fly embryos, with PRC2 subunits detected by immunoblotting, previously characterized PRC2 complexes with apparent molecular weights of 600–700 kDa (25–27). This apparent molecular weight is consistent with PRC2 dimerization, which may exist in addition to the monomeric form that was also observed in human cells by gel filtration (4,28) and was characterized by electron microscopy (EM) (29). Yet, gel filtration cannot exclude the possibility that PRC2 has an extended structure or that the predominant complex has greater molecular mass because it includes additional non-PRC2 proteins, rather than being a dimer.

To address this question we describe here an improved system for the expression and purification of recombinant human PRC2, building on previous PRC2 purification methods using the baculovirus system (4,18,20,24,29–32). Using these reconstituted PRC2 complexes and multiple independent approaches, we provide direct evidence that human PRC2 is a dimer. Specifically, our data support a dimer containing two copies of each subunit. We further show that each PRC2 dimer binds a single RNA molecule .The occurrence of PRC2 as a dimer suggests great diversity among subunit composition of PRC2, considering related isoforms and paralogous protein subunits present in cells. Moreover, a dimeric state of PRC2 has consequences for the molecular mechanism of its binding to molecular recruiters, such as nucleosomes and RNA.

## MATERIALS AND METHODS

### cDNA

pFastBac1 vectors carrying cDNA for the expression of human EZH2, SUZ12, RBBP4 and EED (UniProtKB entry isoform sequences Q15910–2, Q15022, Q09028–1 and O75530–1, respectively) were obtained from Robert Kingston (Harvard Medical School). AEBP2 (UniProtKB Q6ZN18–1) cDNA clone was obtained from OriGene. cDNA clones were fully sequenced before proceeding to subcloning. Before subcloning, synonymous point mutations were introduced in EZH2 and SUZ12 cDNA to remove BsaI restriction sites, allowing for multiparallel cloning using type IIS restriction enzymes (reviewed in (33)), using the following primers:

F primer for removing BsaI site from EZH2: CCATAATGTATTCTTGGTCACCCCTACAGCAGAATTTTATGG

R primer for removing BsaI site from EZH2: CCATAAAATTCTGCTGTAGGGGTGACCAAGAATACATTATGG

F primer for removing BsaI site from SUZ12: CTTCGTTGGACAGGAGAGACGAATGATAAATCTACGGC

R primer for removing BsaI site from SUZ12: GCCGTAGATTTATCATTCGTCTCTCCTGTCCAACGAAG

### Subcloning of vectors for the expression of maltose binding protein (MBP) fusion PRC2 4m and 5m

EZH2, SUZ12, EED, RBBP4 and AEBP2 cDNA were cloned into the pFB1.HMBP vector (derived from the pFastBac1 vector with an N-terminal 6xHis-MBP tag and a PreScission protease cleavage site between MBP and the fused protein). Vectors were digested with BamHI and XbaI. Polymerase chain reaction (PCR)-amplified cDNA was digested with either BsaI (EZH2, SUZ12, EED, RBBP4) or BspMI (AEBP2). Clones were fully sequenced. Primers used for subcloning were as follows:

EZH2 to pFB1.HMBP, forward: GATAGGTCTCGGATCCGGCCAGACTGGGAAGAAATCTG

ybbR-EZH2 to pFB1.HMBP, forward: GATAGGTCTCCCCGGGGATTCTCTTGAATTTATTGCTAGTAAGCTTGCGGGCCAGACTGGGAAGAAATCTG

EZH2 to pFB1.HMBP, reverse: GAATGGTCTCTCTAGATCAAGGGATTTCCATTTCTCTTTCGATG

SUZ12 to pFB1.HMBP, forward: GATAGGTCTCGGATCCATGGCGCCTCAGAAGCA

SUZ12 to pFB1.HMBP, reverse: GAATGGTCTCTCTAGATCACAAATGTGGTATGGCTGATTATG

EED to pFB1.HMBP, forward: GATAGGTCTCGGATCCTCCGAGAGGGAAGTGTCGACT

EED to pFB1.HMBP, reverse: GAATGGTCTCTCTAGATCATCGAAGTCGATCCCAGCG

RBBP4 to pFB1.HMBP, forward: GATAGGTCTCGGATCCGCCGACAAGGAAGCAGCCTTC

RBBP4 to pFB1.HMBP, reverse: GAATGGTCTCTCTAGATCAGGACCCTTGTCCTTCTGGATCCAC

AEBP2 to pFB1.HMBP, forward: GATAACCTGCAGGGGATCCGCCGCCGCTATCACCGACAT

AEBP2 to pFB1.HMBP, reverse: GAATACCTGCCTTTCTAGATCACCTCTTCAACCTCTTCTGCGG

To generate an uncleavable MBP-EZH2 fusion, EZH2 was subcloned into pFB1.HMBP.A3.PrS.ybbR vector (derived from pFastBac1) that was digested with NotI and XhoI. A short linker of three alanine residues connected MBP and EZH2. Cloning was performed using Gibson Assembly (NEB cat # E2611S). Primers used for subcloning were as follows:

EZH2 to pFB1.HMBP.A3.PrS.ybbR (NotI and XhoI), forward: AGACGCGCAGACTGCGGCCGCAGGCCAGACTGGGAAGAAATCTG

EZH2 to pFB1.HMBP.A3.PrS.ybbR (NotI and XhoI), reverse: CTTGGTACCGCATGCCTCGAGTTATCAAGGGATTTCCATTTCTCTTTCGATG

### Cell culture and protein expression

Expression of human PRC2 was carried out as previously described (23), with some modifications. Sf9 cells where used to generate baculovirus stocks using the Bac-to-Bac system (Life Technologies), according to the manufacturer's instructions. Appropriate ratios of baculovirus stocks carrying genes for the four (with the exclusion of AEBP2) or five PRC2 subunits were used to co-infect 4–12 l of Sf9 cells at a density of 2 million cells/ml in Sf-900™ III SFM (Invitrogen cat # 12658–027). Multiple 1 l cell cultures in 4 l flasks (VWR cat # 32645–044) were incubated 72 h at 27°C, 130 revolutions per minute (rpm). Cells were harvested by centrifugation for 20 min at 2000 relative centrifugal force (RCF) (JLA-8.1, Beckman) at 4°C, frozen in liquid nitrogen and stored at −80°C until protein purification.

### Purification of PRC2 expressed using MBP fusion subunits

Sf9 cells (5–80 g) were weighed and thawed. Per 5 g cell paste, 40 ml of cold Lysis Buffer (10 mM Tris-HCl pH 7.5 at room temperature, 250 mM NaCl, 0.5% NP-40, 1 mM TCEP) supplemented with Complete^TM^ protease inhibitor (Roche Diagnostics GmbH, Mannheim, Germany, cat # 11836145001) was added, and cells were gently homogenized and incubated with slow rotation (Rotamix, ATR, Laurel, MD, USA) 10–30 min at 4°C. Lysate was clarified by centrifugation for 30 min at 29 000 RCF (Beckman JA-20 at 15,500 RPM or Beckman JA-17 at 14 500 rpm) at 4°C. All procedures from this step were performed in a 4°C cold room. Per 5 g cell paste, 1.5 ml amylose resin (NEB cat # E8021S) was poured into an Econo-Pac Chromatography Column, 1.5 × 12 cm (Biorad cat # 732–1010) or equivalent column, washed with 4 column volume (c.v.) of double-filtered water and equilibrated with ≥10 c.v. Lysis Buffer. Protein was bound to the amylose resin by passing the lysate through twice. Alternatively, beads were rotated in the lysate for 1–2 h to allow complete binding. Resin was washed with ≥10 c.v. Lysis Buffer and then briefly with 16 c.v. of Wash Buffer 1 (10 mM Tris-HCl pH 7.5 at room temperature, 1000 mM NaCl, 1 mM TCEP). Wash Buffer 1 was immediately removed by washing with 16 c.v. of Wash Buffer 2 (10 mM Tris-HCl pH 7.5 at room temperature, 150 mM NaCl, 1 mM TCEP). Protein was eluted with 3 c.v. of Elution Buffer (Wash Buffer 2 supplemented with maltose (Sigma cat # M5885–100G) to 10 mM) while collecting fractions (0.67 c.v. each). Protein-containing fractions were pooled and concentrated to ∼15 mg/ml (Amicon Ultra-15 Centrifugal Filter Unit, 30 kDa MWCO, Millipore cat # UFC903024). PreScission protease was added at a mass ratio of 1:50 protease:protein and the salt concentration was adjusted to ∼250 mM NaCl. After incubation for 16–20 h at 4°C, cleavage efficiency and specificity were assayed using sodium dodecyl sulphate-polyacrylamide gel electrophoresis (SDS-PAGE) and the protein was injected into HiPrep 16/60 Sephacryl S-500 HR or HiPrep 16/60 Sephacryl S-400 HR, equilibrated with degassed Column Buffer (250 mM NaCl, 10 mM Tris-HCl, pH 7.5 at RT, 1 mM TCEP). Protein was fractionated using a flow rate of 0.5 ml/min. PRC2-containing fractions were identified using SDS-PAGE, pooled and concentrated as above. Protein concentration was determined by absorbance at 280 nm and the absence of nucleic acids was confirmed by ensuring that the ratio of absorbance at 260–280 nm was ≤0.7. Histone methyltransferase (HMTase) activity assays were performed as previously described (23).

### Site-specific fluorescence labeling of PRC2

PRC2 5m was expressed in 1 l of Sf9 culture as described above, except that EZH2 carried an additional ybbR tag (34) immediately downstream from the N-terminal 6xHis-MBP-PreScission tag. EZH2 was N-terminally labeled with CoA 647 fluorophore in parallel to cleavage of the 6xHis-MBP tag. Briefly, eluent from the amylose column was concentrated and adjusted to final concentration of 250 mM NaCl, 10 mM Tris-HCl (pH 7.5 at room temperature), 10 mM MgCl_2_, 2 mM TCEP (pH 7), 0.01 mM CoA 647 (NEB cat # S9350S), 0.054 mg/ml PreScission protease, 0.001 mM recombinant SFP Synthase (plasmid kindly donated by Jun Yin, University of Chicago, protein expressed based on previous work (34)) and 0.005 mM PRC2. The reaction mixture was incubated 16 h at 4°C before being fractionated over a Superdex 200 10/300 column (GE Healthcare) to remove unincorporated fluorophore and cleaved MBP tag.

### Analytical size exclusion chromatography (SEC)

All procedures were carried out at 4°C–8°C. Columns were calibrated using Gel Filtration Standard (BioRad cat # 151–1901) and the void volume was empirically identified using a DNA plasmid (pFastBacDual, Life Technologies cat # 10712–024). Protein samples were injected and fractionated over a Superdex 200 10/300 or Superose 6 PC 3.2/30 column (both GE Healthcare), equilibrated with degassed Column Buffer (10 mM Tris-HCl pH 7.5 at room temperature, 150 mM NaCl). Collected fractions were subjected to SDS-PAGE.

### SEC with multi-angle light scattering (SEC-MALS)

All procedures were carried out at 25°C. Protein samples were filtered (Ultrafree-MC Centrifugal Filter Units with Microporous Membrane, 0.1 micron, Millipore cat # UFC30VV00) and injected into Shodex Protein KW-803 column, equilibrated with 0.1 micron filtered and degassed Column Buffer (150 mM K_2_SO_4_, 10 mM Tris-HCl pH 7.5 at room temperature). Mobile phase was subjected to MALS using a Wyatt Dawn EOS 18-angle light scattering detector and Wyatt Optilab DSP refractive index monitor. Calibration was performed using protein standards (BioRad cat # 151–1901, Pierce cat # 23209, Sigma cat # A8656–1VL).

### Templates for *in vitro* transcription

The first 400 bp of the human HOTAIR gene (RefSeq accession NR_003716.2) were obtained by gene synthesis (plasmid H001, for sequence see (23)). PCR primers for the amplification of DNA templates for *in vitro* transcription were designed using DNADynamo (Blue Tractor Software) and synthesized by Integrated DNA Technologies (IDT). DNA templates for *in vitro* transcription of HOTAIR 1–200 RNA were amplified from the plasmid H001 using primers with the following sequences:

T7-F: TAATACGACTCACTATAGGGAGACATTCTG

R: GGTCTAAGTCCCGGGTGGGA

DNA template for *in vitro* transcription of the full length HOTAIR RNA was generated from plasmid LZRS-HOTAIR (AddGene plasmid 26110 (35)) using the following primers:

T7-F: TAATACGACTCACTATAGGGAGACATTCTGCCCTGATTTCCGG

R: TTTTTTCACCACATGTAAAACTTTATTTATGCATAAAAC

### Electrophoretic mobility shift assay (EMSA) for PRC2-RNA binding

*In vitro* RNA transcription and radiolabeling were carried out as previously described (23). Radiolabeled RNA, 500–2000 cpm with specific activity no less than 100 000 cpm/pmol, was adjusted to a volume of 4.3 microliter with Milli-Q pure water. RNA was incubated 1 min at 95°C and snap-cooled on ice for 2 min. RNA was then allowed to fold for 30 min at 37°C in Binding Buffer (50 mM Tris-HCl pH 7.5 at 25°C, 100 mM KCl, 5 mM MgCl_2_, 0.5 mM ZnCl_2_, 0.1 mM CaCl_2_, 2 mM 2-mercaptoethanol, 0.1 mg/ml bovine serum albumin, 0.1 mg/ml fragmented yeast tRNA (Sigma cat # R5636), 5% v/v glycerol, 0.025% w/v bromophenol blue and 0.025% w/v xylene cyanol). Next, protein was added and allowed to bind at 30°C in Binding Buffer for 30 min. Sample was cooled to 4°C before loading on a 10–15 cm horizontal non-denaturing 0.7% agarose gel (SeaKem® GTG® Agarose, Fisher Scientific cat # BMA 50070) buffered with 1XTBE at 4°C. Gel electrophoresis was carried out for 90 min at 6.6 V/cm in an ice box within a 4°C cold room, unless otherwise indicated. Gels were vacuum dried for 60 min at 80°C on a nylon membrane and two sheets of Whatman 3 mm chromatography paper. Dried gels were exposed to phosphorimaging plates and signal acquisition was performed using a Typhoon Trio phosphorimager (GE Healthcare). When CoA 647 fluorescently labeled protein was included, bromophenol blue and xylene cyanol dyes were substituted by orange G and gels were scanned for fluorescence before drying.

### EMSA to identify the number of RNA molecules bound to PRC2

This experiment was carried out as described above, except that unlabeled RNA was included also in the reactions (for detailed description see Results section). In order to distinguish between different complexes and unbound RNAs that were present within reaction mixtures, gel electrophoresis conditions were optimized and agarose-acrylamide composite gels were used. Specifically, agarose (SeaKem® GTG® Agarose, Fisher Scientific cat # BMA 50070) was microwaved in 50 ml 1x Tris-Glycine, pH 8.3 (Invitrogen cat # LC2672) to yield a 1.4% (w/w) solution. Melted agarose was equilibrated in a 45°C water bath before mixing with 50 ml of 2% 29:1 acrylamide:bis solution, buffered with 1x Tris-Glycine, pH 8.3. 0.5 ml of 10% (w/w) ammonium persulfate and 0.05 ml TEMED were added and solution was mixed before pouring to form a composite 0.7% agarose/1.0% acrylamide gel. The gel was allowed to solidify at least 1 h at room temperature, to allow both the agarose and acrylamide to polymerize, before it was submerged in 1x Tris-Glycine, pH 8.3, running buffer and stored up to 3 days at 4°C before use. Horizontal submerged gel electrophoresis was carried out for 2 h at 100 V over 10 cm gels at 4°C. Gels were vacuum dried over a positively charged membrane (Hybond-N+, GE Healthcare cat # RPN203B) for 2 h at 80°C following by 15 min at room temperature before releasing the vacuum and transferring into a phosphorimager cassette.

### Titration experiment to identify binding stoichiometry of PRC2 to RNA

Titration experiments to identify PRC2-RNA binding stoichiometry (36) were carried out as described above for direct binding assays, but instead of using a trace amount of radiolabeled RNA, non-radiolabeled RNA was added to a final concentration of 50–200 nM. Titration was performed over a range of 2-fold PRC2 5m dilutions, starting from 1600 nM. Densitometry was carried out as above, and the concentration of bound RNA was calculated by multiplying the fraction of bound RNA by the total RNA concentration. Titration with two different RNA concentrations independently ensured that the RNA concentration was sufficiently high to achieve saturation.

### Co-immunoprecipitation

PRC2 complexes were expressed in Sf9 cells and purified as nucleic acid-free complexes as described above. Three different recombinant human PRC2 5m complexes were purified: (i) untagged PRC2 5m (EZH2, SUZ12, EED, RBBP4 and AEBP2), (ii) MBP tagged PRC2 complex (MBP-EZH2, SUZ12, EED, RBBP4 and AEBP2) and (iii) PRC2 5m complex including both tagged and untagged EZH2 (MBP-EZH2, EZH2, SUZ12, EED, RBBP4 and AEBP2). To ensure compatibility with the purification procedure, all co-expressed PRC2 subunits had a cleavable PreScission tag following the N-terminal MBP fusion; the exception was EZH2, which had an uncleavable MBP tag in the case where an MBP tag was required post-purification.

For each complex, 0.1 mg PRC2 was dissolved in 0.2 ml Binding Buffer (10 mM Tris-HCl pH 7.5, 250 mM NaCl and 1 mM TCEP) and applied to 0.05 ml amylose beads (NEB cat # E8021S), pre-equilibrated with Binding Buffer. Protein was allowed to bind beads for 30 min at 4°C in a 0.5 ml spin column (Spin Cups, Pierce cat # 69700). Unbound protein was then removed by centrifugation (30 s at 500 RCF, 4°C) and beads were subsequently washed twice with 0.5 ml ice cold Binding Buffer. Beads were then resuspended in 0.075 ml Elution Buffer (Binding Buffer, supplemented with maltose to 20 mM). Beads were incubated 10 min at 4°C before the eluent was released into a clean tube by centrifugation (30 s at 500 RCF, 4°C). For SDS-PAGE, eluted proteins were denatured by adding 0.025 ml of 4X NuPAGE® LDS Sample Buffer (Life Technology cat # NP0007), supplemented with 2-mercaptoethanol to 4% v/v, followed by 5 min incubation at 95°C. For immunoblotting, samples were further diluted 1:1 with 1X NuPAGE® LDS Sample Buffer (Life Technology cat # NP0007), supplemented with 2-mercaptoethanol to 1% v/v. For semi-quantitative immunoblotting, several 3-fold dilutions were prepared from each sample by pipetting 0.008 ml sample (already in 1X NuPAGE® LDS Sample Buffer) into 0.016 ml of 1X NuPAGE® LDS Sample Buffer. Portions (0.01 ml) of the input and eluent diluted samples were loaded on a 4%–12% Bis-Tris NuPAGE SDS gel (Invitrogen cat # NP0322BOX), run at 150 V for 45 min and transferred to Hybond ECL membrane (GE lifesciences cat # RPN78D) for immunoblotting. Antibody details and titers used for immunoblotting are indicated in Supplementary Table S1.

## RESULTS

### Expression and purification of PRC2 4m and PRC2 5m as MBP fusions

Previous work showed that human PRC2 5m does not have a compact structure and that the N-termini of its subunits lie close to the surface of the complex (29). Therefore, we anticipated that fusing a large N-terminal tag to all subunits simultaneously would not prevent complex assembly, as each fused tag would have adequate space to be accommodated. Thus, in order to increase expression yield and protein solubility we co-expressed all subunits with N-terminal *Escherichia coli* MBP tags; the MBP tag is known to be effective at promoting high expression level and solubility of its fused partners (37). The rationale of fusing MBP to all subunits simultaneously was to surround PRC2 with a temporary artificial shell of well-folded proteins. This protecting shell was anticipated to shield PRC2 from undesired interactions with partially unfolded subunits or cellular proteins and nucleic acids in the host insect cell and in the lysate. A cleavable PreScission tag included downstream from each MBP fusion allowed complete removal of the multiple-MBP protecting shell after the complex was isolated from the lysate.

**Figure 1.**
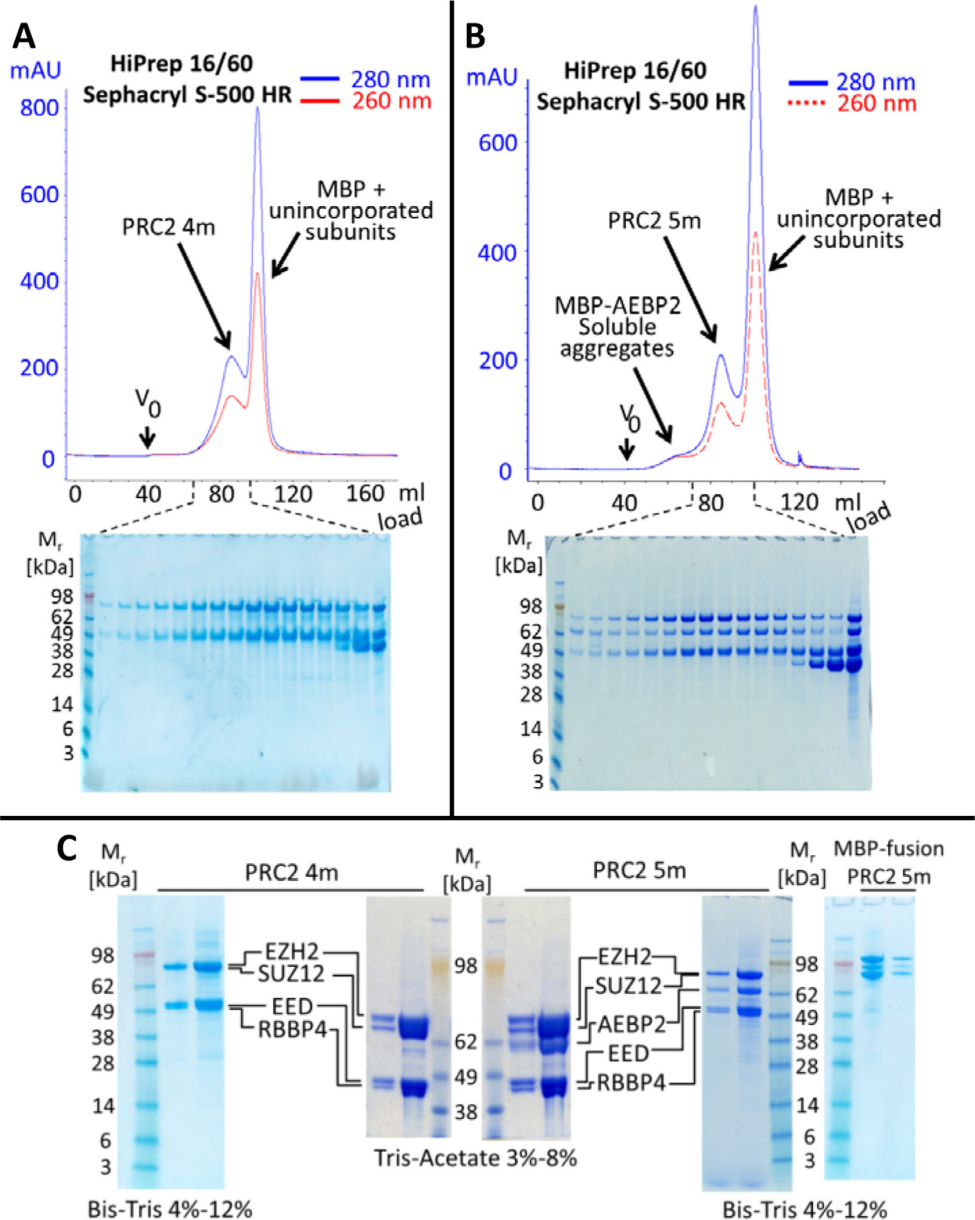
Expression and purification of PRC2 4m and PRC2 5m as MBP fusions. (A) Fractionation of PRC2 4m over Sephacryl S-500 HR column after cleavage of MBP tags. (B) Identical process performed for PRC2 5m. (C) SDS-PAGE confirms that all PRC2 subunits appear with the expected molecular weights, before and after cleaving MBP tags.

Chromatograms and SDS-PAGE of recovered fractions confirmed separation of the assembled complex from unincorporated subunits, cleaved MBP tags and soluble aggregates (Figure 1A and B). The presence of all subunits in the complex and the absence of unidentified proteins were assayed using SDS-PAGE (Figure 1C). This was further confirmed by comparing samples before and after cleavage by PreScission protease (MBP-PRC2 5m prior to cleavage shown in Figure 1C, far right). PRC2 purity and subunit identity were confirmed by mass spectrometry (Supplementary Table S2) and catalytic activity *in vitro* was confirmed by a histone methyltransferase assay (Supplementary Figure S1), with PRC2 5m having higher activity than PRC2 4m as previously reported (4). Affinity purification using amylose resin is scalable and relatively economical. Accordingly, this purification scheme allowed us to obtain assembled and soluble PRC2 in amounts required for the performance of multiple quantitative EMSA assays (23).

### Reconstituted PRC2 is a dimer

**Figure 2.**
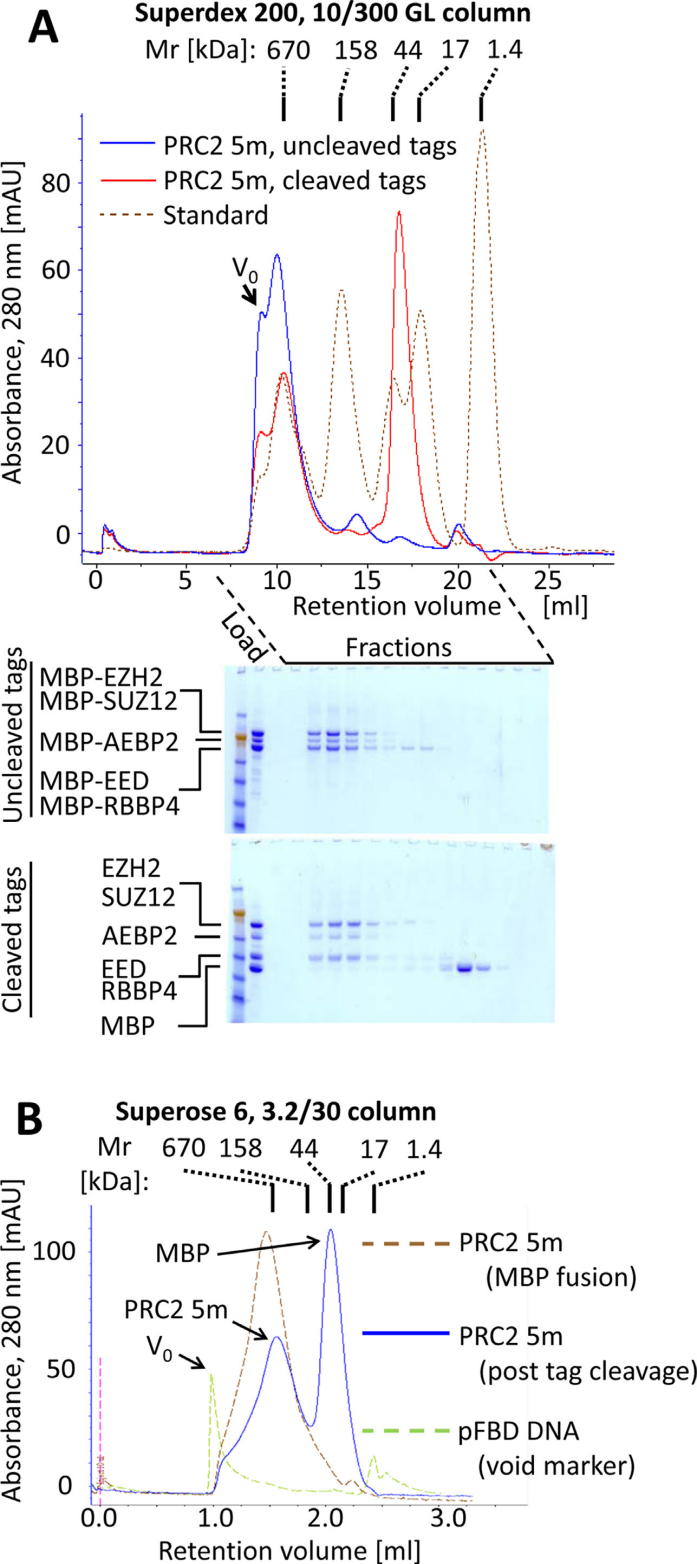
SEC confirming that reconstituted PRC2 5m is a dimer. (A) SEC using Superdex 200 10/300 column for PRC2 5m, before and after cleaving MBP tags. (B) SEC using analytical Superose 6 PC 3.2/30 column for PRC2 5m, before and after cleaving MBP tags.

To test whether the purified PRC2 had its expected molecular weight, we loaded the complex, before cleaving the MBP tags, on a calibrated Superdex 200 10/300 column. Under the assumption of a stoichiometrically assembled complex, including a single copy of each subunit, the molecular weight of MBP-PRC2 5m is predicted to be 540 kDa (Table 1). Yet, the complex eluted earlier, with a retention volume representing an apparent molecular weight larger than 670 kDa (Figure 2A). We repeated this experiment after cleavage of the MBP tags (Figure 2A), but still the complex failed to resolve on the column, suggesting a molecular mass much higher than that calculated for PRC2 as a monomer (320 kDa, Table 1). To gain better separation of complexes in this size range, we repeated this experiment using Superose 6 resin packed in an analytical Precision Column PC 3.2/30, before and after cleaving MBP tags (Figure 2B). Upon cleavage of MBP tags the retention volume of the complex increased, representing an apparent molecular weight of ∼670 kDa, about twice the expected size of PRC2 5m (320 kDa, Table 1).

**Table 1.** Molecular weights of PRC2 subunits and complexes. Molecular weights (MWs) for PRC2 subunit proteins presented as expressed in the scope of this study and are identical to MWs of the indicated UniProt IDs. MWs for PRC2 complexes in different oligomerization states, in the presence or absence of MBP tags, are considering one copy of each protein subunit within PRC2 monomer and two copies in a dimer.

Protein	MW [kDa]	UniProt ID
EZH2	85	Q15910–2
SUZ12	83	Q15022
AEBP2	54	Q6ZN18–1
EED	50	Q09028–1
RBBP4	48	O75530–1
MBP*	44	P0AEX9
PRC2 4m	266	n.a.
PRC2 5m	320	n.a.
MBP-PRC2 4m	442	n.a.
MBP-PRC2 5m	540	n.a.
PRC2 4m dimer	533	n.a.
PRC2 5m dimer	641	n.a.
MBP-PRC2 4m dimer	884	n.a.
MBP-PRC2 5m dimer	1080	n.a.

*Molecular weight of MBP includes 6xHis and PreScission tags.

**Figure 3.**
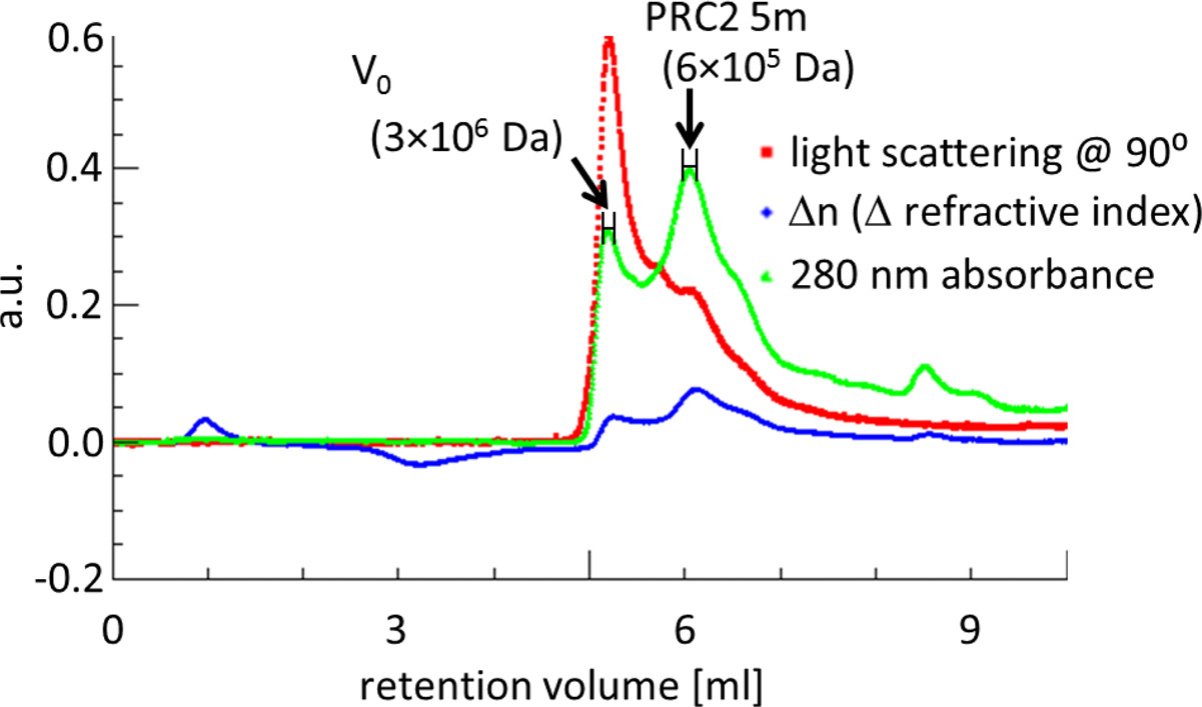
SEC-MALS confirming that reconstituted PRC2 5m is a dimer. SEC-MALS performed using Shodex Protein KW-803 column for PRC2 5m, after removing MBP tags. Retention volume range used to calculate molecular mass is shown for each peak.

While this result suggested that PRC2 is a dimer, it seemed possible that retardation of the complex in these two different sizing columns might be caused by an unusually extended conformation, rather than representing an increased molecular weight. We therefore subjected the complex to SEC-MALS, because this approach is capable of measuring the molecular weight of a given complex independent of its conformation (38). The main peak, based on absorbance at 280 nm, corresponded to 6·10^5^ Da (Figure 3), in good agreement with our independent SEC experiments (Figure 2).

To test the oligomerization state of PRC2 using an additional independent approach, we designed a pull-down experiment using PRC2 5m, where a mixture of tagged and untagged EZH2 was allowed to incorporate into the complex. If PRC2 were a dimer, we anticipated that both tagged and untagged EZH2 would track within the same complex. To this end, we expressed human PRC2 5m in the baculovirus system with EZH2 carrying a mixture of cleavable and uncleavable MBP tags within the same batch. All other subunits had cleavable MBP tags (for full description see Materials and Methods section above). Thus, following Precision protease treatment only MBP-EZH2 with an uncleavable linker retained an MBP tag. As the presence of RNA contaminants could potentially stabilize PRC2 dimers artificially *in vitro*, the purification scheme eliminated nucleic acid contaminants (see Materials and Methods) as evidenced by maintaining a low ratio of absorbance at 254 nm to 280 nm (less than 0.7, see Supplementary Figure S2 for complete fast protein liquid chromatography trace for this complex).

**Figure 4.**
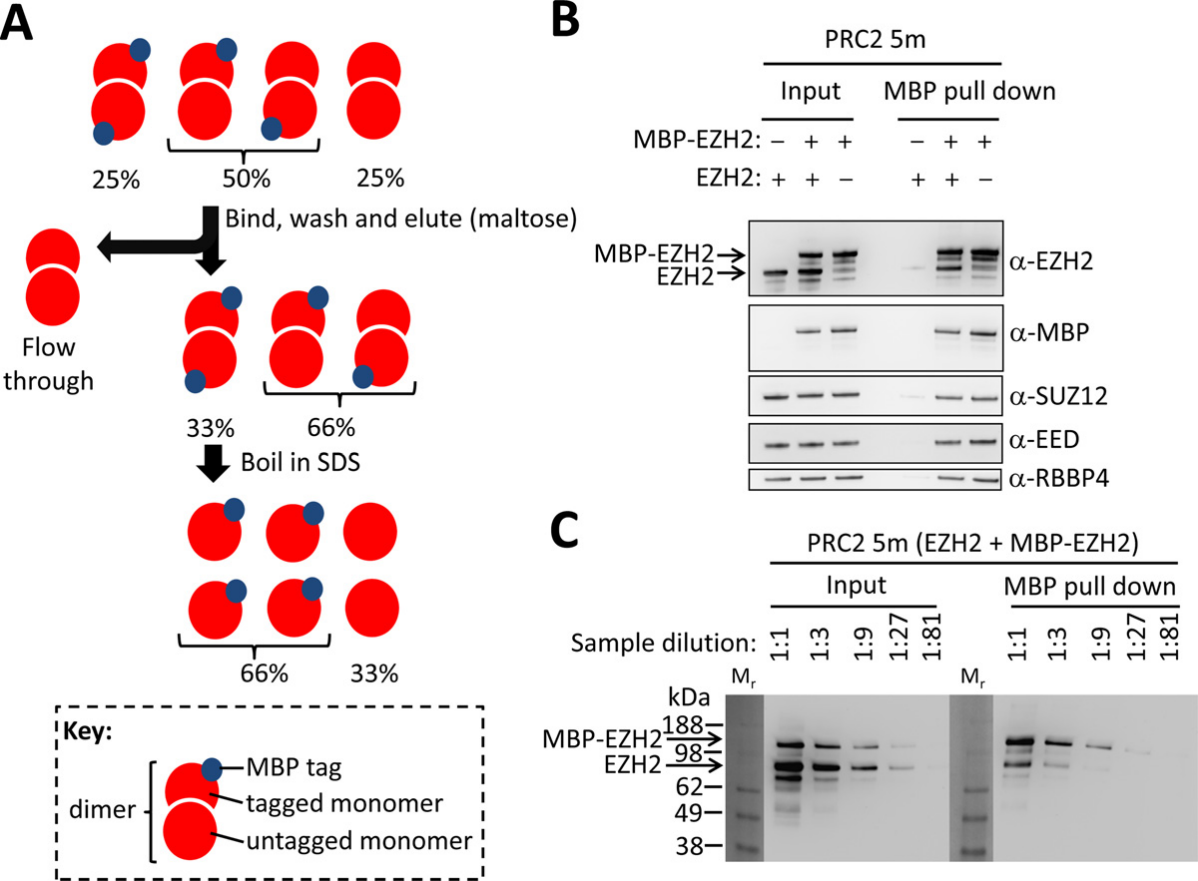
Co-immunoprecipitation indicates the existence of more than one EZH2 within reconstituted PRC2. (A) Experiment outline: nucleic acid-free human PRC2 was reconstituted by co-expressing and co-purifying EZH2 as both MBP-tagged and untagged variants, together with all four other subunits as untagged proteins. Complexes containing at least one copy of tagged EZH2 were bound to amylose beads, washed and eluted. The expected fraction of each variant in each step is indicated for the case of dimeric PRC2. (B) Input, sample prior to MBP pull down. MBP pull down, complexes containing MBP-EZH2 were recovered using amylose beads. Immunoblotting using an anti-EZH2 antibody confirmed the presence of both MBP-tagged and untagged EZH2 in the complex. Controls included the same complex reconstituted either without untagged EZH2 or without MBP-tagged EZH2, as indicated. Immunoblotting using an anti-MBP antibody confirmed the presence of the MBP tag only on designated samples and the absence of C-terminal truncated MBP-EZH2 with molecular weight similar to EZH2. Immunoblotting was used to confirm the presence of other core PRC2 subunits SUZ12, EED and RBBP4 (see Supplementary Figure S3 for complete blots). (C) Semi-quantitative immunoblotting using anti-EZH2 antibodies revealed a ratio of ∼3:1 tagged:untagged EZH2 in the MBP pull-down sample.

Cells were lysed and complexes containing MBP-EZH2, either in the presence or absence of untagged EZH2, were captured on amylose resin. Washed beads were subjected to specific elution using maltose. In a perfect experiment, one would expect to obtain a ratio of 2:1 tagged:untagged EZH2 within the MBP pull down for dimeric PRC2 (Figure 4A). This is under the assumption that no dimers dissociate during the washes and that the double-tagged dimer is not preferentially bound to the beads. A notable strength of this assay is that even if the untagged version of EZH2 is present in excess over the tagged version within the input, one should expect the ratio of tagged:untagged EZH2 to be no lower than 1:1, because the excess untagged complexes will not bind the beads.

On the other hand, if PRC2 were a monomer, one would expect to obtain only the tagged version of EZH2, as in the absence of dimerization the untagged EZH2 would be washed away. However, immunoblotting with anti-EZH2 antibody confirmed the presence of both MBP-EZH2 and untagged EZH2 in the MBP pull down (Figure 4B). Semi-quantitative immunoblotting identified that EZH2 tagged:untagged ratio was ∼3:1 (Figure 4C). This ratio is somewhat higher than the 2:1 tagged:untagged ratio expected for a dimer, but yet it clearly confirms the presence of both tagged and untagged EZH2 within the same complex.

To exclude the possibility that the untagged EZH2 was carried by the beads through non-specific interactions, we repeated this experiment with PRC2 5m complex carrying no MBP tags (Figure 4B). To discount the possibility that a band with the same molecular weight as the untagged EZH2 was obtained because of undesired proteolysis of MBP-EZH2, we repeated the experiment with a complex including only MBP-EZH2, with no untagged EZH2 (Figure 4B). Both controls showed little untagged EZH2 in the MBP pull down, compared with the sample containing both tagged and untagged EZH2.

We conclude that there are two or more EZH2 molecules per 5m complex, consistent with our observation of a dimer using SEC and SEC-MALS. Based on the apparent molecular weights that were obtained in our SEC (Figure 2) and SEC-MALS experiments (Figure 3), the PRC2 5m dimer is proposed to contain two molecules of each of the five subunits.

### Each PRC2 dimer interacts with a single RNA molecule

**Figure 5.**
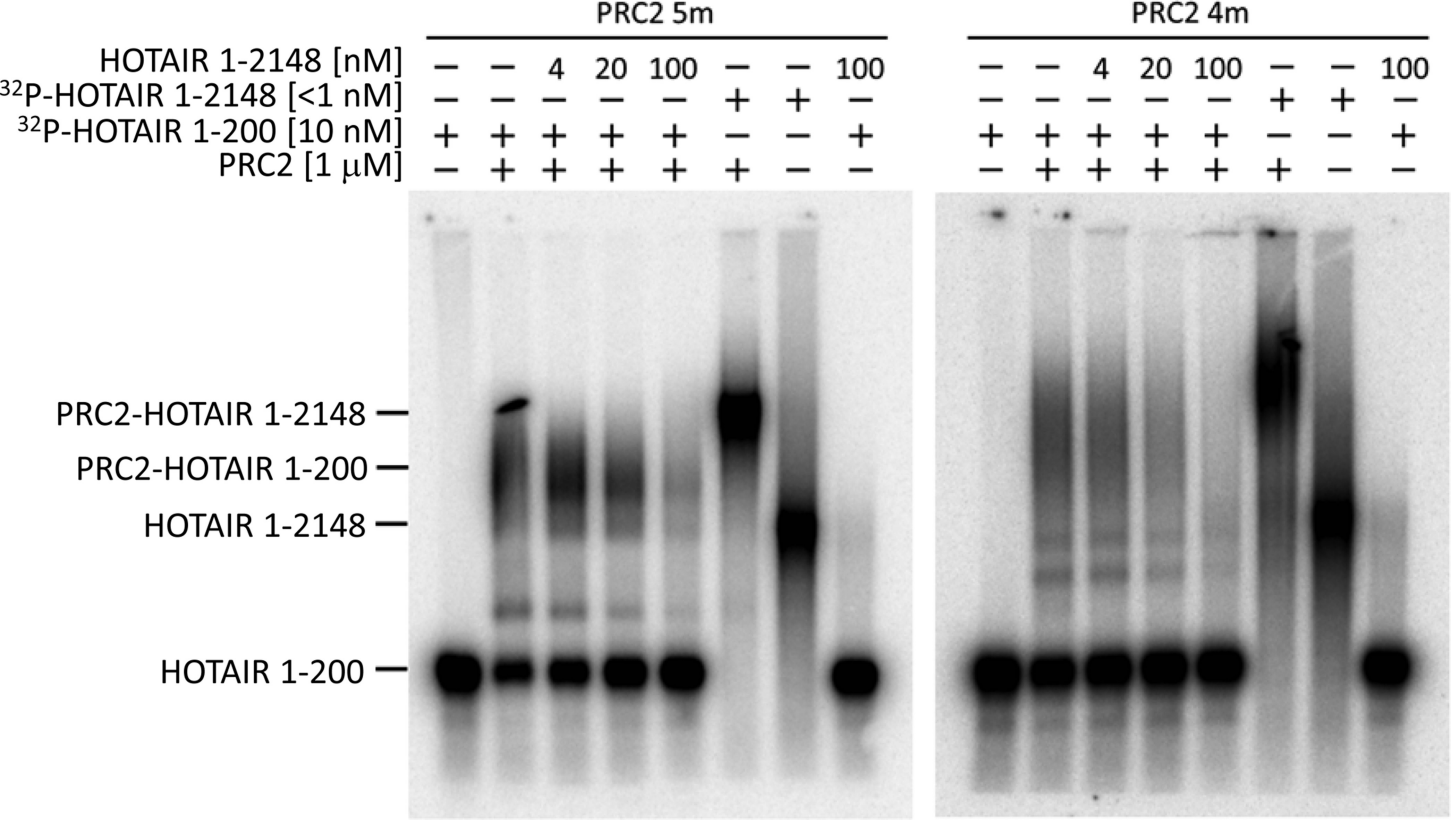
Each PRC2 dimer interacts with a single RNA molecule. PRC2 5m or 4m was incubated with a low concentration of radiolabeled HOTAIR 1–200 and various concentrations of unlabeled HOTAIR 1–2148 and then subjected to EMSA. The electrophoretic mobility of PRC2-HOTAIR 1–200 was identical in the presence or absence of the unlabeled HOTAIR 1–2148, indicating that HOTAIR 1–2148 is not incorporated into the PRC2-HOTAIR 1–200 complex. A notable retardation of PRC2 was obtained when bound to HOTAIR 1–2148 alone, in the absence of HOTAIR 1–200, demonstrating the ability of these EMSA conditions to detect such a supershift, if it were present.

Different studies have reported EZH2 or SUZ12 to be the RNA-binding subunit of PRC2 (18,19,24). Given that PRC2 is a dimer, it must contain two (or more) RNA-binding subunits. We therefore tested whether a single PRC2 dimer can bind two RNAs simultaneously, or if the PRC2 dimer is constrained to bind one RNA molecule at a time. We performed EMSA in the presence of PRC2 and two different RNA ligands: short RNA (HOTAIR 1–200, 205 bases) and long RNA (HOTAIR 1–2148, 2153 bases). Electrophoresis conditions were optimized to allow discrimination of the four different bands comprised of the short and long RNAs unbound or bound to PRC2 (Figure 5). We incubated PRC2 in the presence of the short radiolabeled RNA and increasing amounts of unlabeled long RNA. Notably, the migration distance of the band representing the complex of PRC2 and the short RNA remained constant, independent of the concentration of the unlabeled long RNA. The lack of a supershift indicates that complexes including both short and long RNAs never occurred. Instead, when the concentration of the unlabeled long RNA was increased it competed off the short RNA from PRC2, indicating that each PRC2 dimer interacts with a single RNA molecule. Given the equivalent results for PRC2 4m and 5m, this conclusion holds both in the presence and absence of AEBP2.

### A single RNA molecule can bind multiple PRC2s

**Figure 6.**
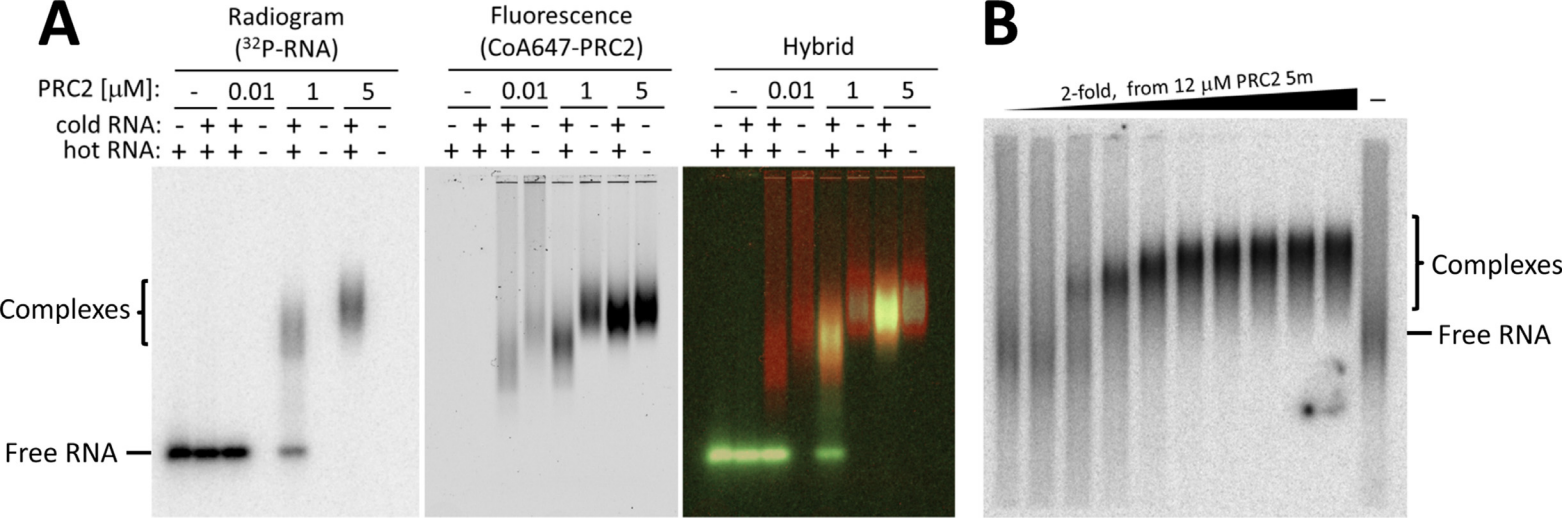
A single RNA molecule can bind multiple PRC2s. (A) Different concentrations of PRC2 5m were incubated with 10 nM fluorescent PRC2 and 500 nM radiolabeled HOTAIR 1–200 RNA. Retardation of PRC2-RNA complex was observed upon increasing the PRC2 concentration, indicating multiple PRC2 binding events on the RNA. (B) Different concentrations of PRC2 5m were incubated with HOTAIR 1–2148, before performing EMSA. In addition to the expected increment in the fraction of bound RNA, gradual retardation of HOTAIR 1–2148 occurred with increasing PRC2 concentration, indicating multiple binding events of PRC2 on the RNA.

To test whether a single RNA molecule can bind multiple PRC2 complexes, we performed EMSA using different concentrations of PRC2 5m, spiked with trace amount of fluorescently labeled PRC2, in the presence of a saturating amount (500 nM) of radiolabeled HOTAIR 1–200 RNA. Radioactive signal from the RNA and fluorescence from the protein were monitored from the same gel, which allowed identification of the location of both the RNA and the protein in the gel (Figure 6A).

Importantly, PRC2 was almost completely shifted in the presence of RNA, indicating that the fraction of protein that is active in RNA binding is high. This suggests that the apparent equilibrium dissociation constants that we previously measured to quantify the affinity of PRC2 to RNA (23) approximately equal the absolute dissociation constants. As expected, RNA increased the mobility of PRC2 in the gel, resulting in faster migrating bands in all RNA-containing lanes. Yet, retardation of the RNA-protein complex occurred with increased protein concentration, suggesting that a single RNA molecule binds multiple PRC2s.

We further performed EMSA for PRC2 5m in the presence of a long RNA (HOTAIR 1–2148). We assumed this RNA to be sufficiently long to provide multiple binding sites given that PRC2 is capable of binding RNA promiscuously *in vitro* (23) and *in vivo* (23,39) and that an RNA of 25 bases is sufficiently long for binding (23). The resulting radiogram revealed RNA retardation in a PRC2 concentration-dependent manner (Figure 6B), indicating multiple PRC2 binding events on the same RNA. In agreement with these findings, a titration experiment confirmed an average of 1.7 bound PRC2 dimers, or 3.4 bound monomers, to MBP 1–200 RNA (Supplementary Figure S4), an RNA that was previously shown to bind PRC2 without a well-defined binding motif (23).

## DISCUSSION

Here we provide evidence that reconstituted, catalytically active and nucleic acid-free human PRC2 is a dimer. This conclusion is consistent across analytical SEC (Figure 2), SEC-MALS (Figure 3) and co-immunoprecipitation experiments (Figure 4). Endogenous PRC2 complexes from both human and fly were previously characterized in multiple studies using SEC and found to have a wide range of apparent molecular weights, including 300–400 kDa (4,28,29), 600–700 kDa (25–27), 790 kDa (40) and 1 MDa or higher (26–28,40). The large variety of molecular weights that were previously observed for PRC2 indirectly suggests that the complex can adopt different oligomerization states, in addition to the monomeric state that was recently characterized by electron microscopy (29). Yet, these previously obtained results by themselves could have been subject to other interpretations. For instance, the majority of these studies characterized endogenous PRC2, rather than a recombinant complex. Therefore, it was difficult to assess if the high molecular masses were due to a higher oligomerization state or to uncharacterized proteins or nucleic acids that were associated with the complex. A recent report identified monomers, dimers, trimers and higher order oligomers of PRC2 3m (EZH2-EED-SUZ12) (41), as opposed to the discrete dimeric state observed here for PRC2 4m and 5m. One concern is that a PRC2 3m complex might have an exposed binding surface due to the absence of the RBBP4 subunit, thereby leading to partial aggregation. Clearly, it will be important to test for the presence of endogenous PRC2 dimers in human cells in the absence of overexpression.

We find that each PRC2 dimer interacts with a single RNA molecule (Figure 5). Assuming that each PRC2 monomer within the dimer contributes an RNA-binding site, then binding of a single RNA molecule by a PRC2 dimer will require having two binding sites for PRC2 on the RNA ligand. We have recently shown that PRC2 binds RNA promiscuously, without requiring a specific motif (23). Therefore, binding of RNA to one site would consequently result in binding of another portion of the same RNA to the second site, driven by the increased local concentration of the RNA and the lack of a strict requirement for a specific RNA sequence signature. In such case, having PRC2 as a dimer is predicted to increase the binding enthalpy by twice the expected value for a monomer (42). Considering an RNA binding constant on the order of 100 nM for a PRC2 dimer (23) (Figure 1), the PRC2 monomer would be expected to bind RNA with millimolar affinity, which is biologically irrelevant. Thus, PRC2 dimerization allows it to bind RNA promiscuously with submicromolar affinity. One corollary of this model is that an RNA too short to span between subunits should not engage in stable binding (i.e. it would have only millimolar affinity). This is entirely consistent with our previous study of the RNA length dependence of binding, wherein a 15 nt RNA showed no binding but RNAs of 25 nt or longer bound with good affinity (23).

**Figure 7.**
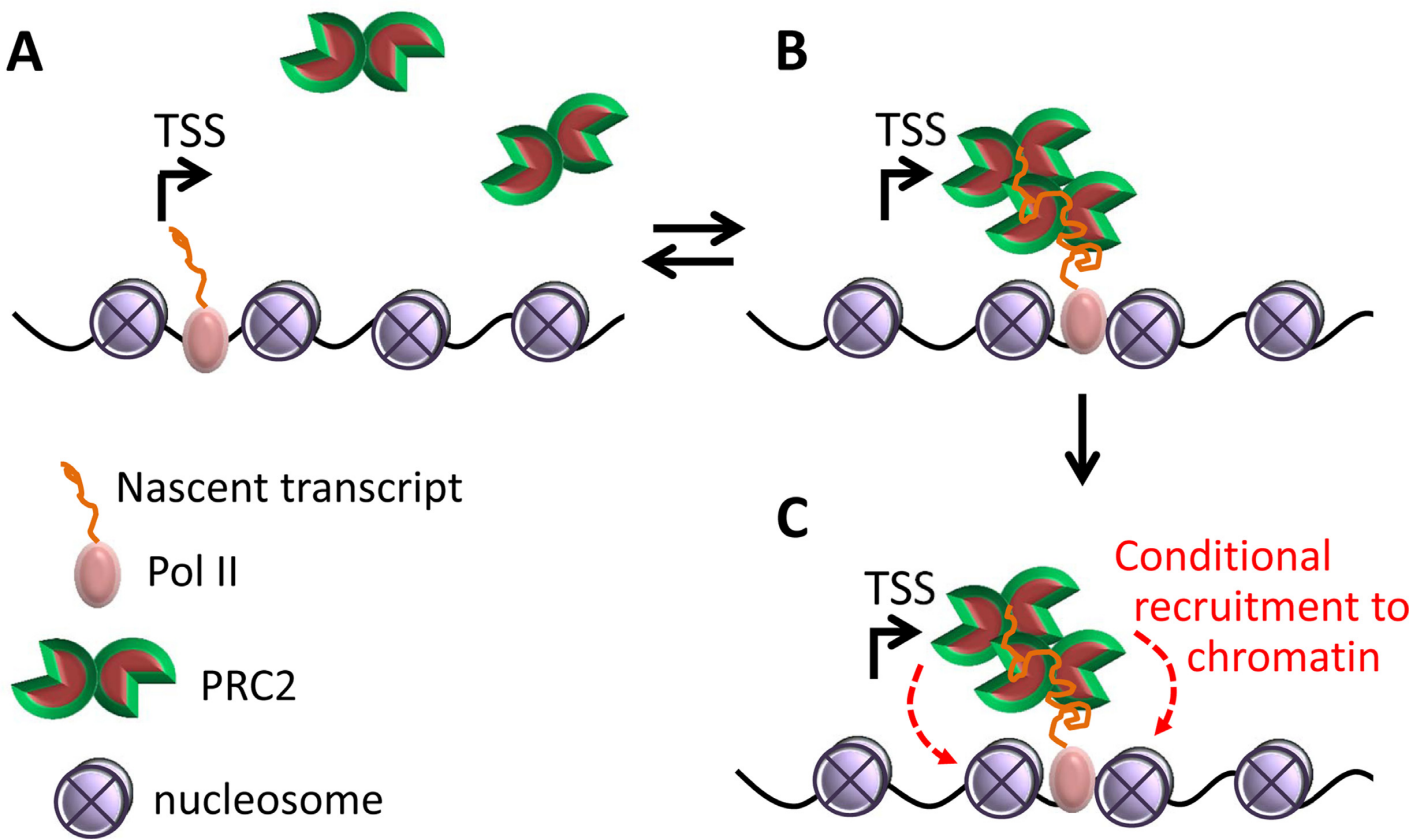
Molecular model for the recruitment of multiple PRC2s following a single RNA binding event. (A) Short nascent transcripts are unlikely to bind PRC2, as the affinity of PRC2 to short RNAs is low (23). (B) When a nascent transcript reaches a length of a few tens to a few hundreds of bases, sufficient to span both RNA-binding sites on a PRC2 dimer, then PRC2 can bind the RNA promiscuously with submicromolar affinity (23). Binding involves multiple PRC2 dimers that bind the RNA in a cooperative manner, thus providing a potential nucleation point for recruitment to chromatin. (C) If PRC2 encounters previously deposited H3K27me3 marks, its affinity to nucleosomes will increase (5,6,44) and it will deposit to chromatin as shown. On the other hand, if PRC2 senses H3K4me3 or H3K36me3 active marks, its affinity to nucleosomes will be reduced (7,8), preventing recruitment to chromatin. In the absence of recruitment to chromatin, the RNA may serve as a decoy that will strip PRC2 away from active genes (23,39).

While each PRC2 dimer binds a single RNA, our data indicate multiple binding events of PRC2 dimers to a single RNA molecule (Figure 6 and Supplementary Figure S4). This agrees well with the lack of a strict requirement for a specific RNA sequence signature (23,39), which allows for multiple binding sites for an RNA that is sufficiently long. A single binding event of a pre-formed protein dimer is not expected to result in any binding cooperatively (36). We have previously observed that PRC2 binds RNA cooperatively (23). This indirectly suggests that the first stable RNA-protein complex involves more than a single PRC2 dimer. While the actual binding stoichiometry cannot be determined merely from the Hill coefficient in the case of multiple binding sites on an RNA ligand (43), in the case of positive cooperativity the initial recruitment event already results in a large multiprotein complex, including at least four PRC2 monomers; any subsequent binding event (Figure 6 and Supplementary Figure S4) will further increase the number of PRC2 complexes per RNA. This process may result in a nucleation point for PRC2 recruitment to chromatin (Figure 7). If the H3K27me3 mark is present on chromatin, the affinity of PRC2 to nucleosomes increases and it will be deposited to nucleosomes (5,6,44). In the presence of an active mark—H3K4me3 or H3K36me3—the affinity of PRC2 to nucleosomes is low (7,8), thus recruitment to chromatin is unlikely to occur, as previously suggested based on the Junk Mail Model (23). This model ensures that when the appropriate conditions are available, multiple PRC2 complexes can be deposited from the nascent transcript to nucleosomes at the recruitment site, boosting the repression process (Figure 7). This model requires further validation, ideally by the establishment of PRC2 mutants or variants that are incapable of dimerization but maintain their HMTase activity.

Another potential biological significance of PRC2 dimerization could be an ability to bridge adjacent nucleosomes by binding to two H3 tails simultaneously, thereby aiding in chromatin compaction or giving PRC2 higher affinity for already-compacted chromatin. Such a mechanism was recently demonstrated for PRC1, a histone ubiquitin ligase that associates with the H3K27me3 mark deposited by PRC2. Accordingly, PRC1 H3K27me3 binding combined with oligomerization of the SAM domain of the complex core component Phc2 was suggested to facilitate subnuclear clustering during epigenetic repression (45). This would also be analogous to *Schizosaccharomyces pombe* HP1 (Heterochromatin Protein 1) dimers, which can bridge nearby nucleosomes by binding methylated H3K9 tails *in vitro* (46); this has been proposed to stabilize heterochromatin (47). The affinity of the PRC2 subunit EED for repressive mark histone tail peptides H3K27me3, H3K9me3 and H1K26me3 lies within the mid-micromolar range (*K*_d_ > 20 μM (5,6)). Although significantly higher than affinities to unrelated histone tail peptides, these affinities seem too low to confer biological relevance *per se*. However, if two histone tails are conjugated, for instance by occurring on adjacent nucleosomes or the same nucleosome, then the affinity for a PRC2 dimer would be greatly enhanced (42).

In principle, PRC2 dimerization can result in a large variety of complexes. Thus, each of the human PRC2 subunits, with the exception of SUZ12, has several known isoforms resulting from different splice variants. Two subunits, namely, EZH2 and RBBP4, have paralogous proteins in mammals. EZH1 (48) is a functionally distinguished paralog of EZH2 that can be incorporated into PRC2 (25,49,50). RBBP4 and RBBP7 are two paralogous proteins appearing in mammals and can both be present in the complex (reviewed in (1)). Therefore, PRC2 dimers can potentially be assembled with a variety of subunit combinations, considering all known paralog subunits and isoforms, resulting in functional variations. The composition of PRC2 complexes can further vary between cells and through differentiation. For instance, the ratio of the expression level of EZH2 to EZH1 varies between cell types (50,51), which may lead to variations in PRC2 subunit composition. Interestingly, a previous study identified co-association of EZH2 and EZH1 using co-immunoprecipitation (25), suggesting the presence of both proteins within the same complex (52). Another source of variation in PRC2 subunit composition can occur in the process of X chromosome inactivation, which takes place during embryonic development in females (reviewed in (53)). Remarkably, in mammals RBBP7, but not RBBP4, is encoded by the X chromosome. Therefore, it is possible that the ratio of expression level of these two paralogous PRC2 subunits is being altered throughout the process of X chromosome inactivation.

While the actual function of PRC2 dimerization *in vivo* has yet to be elucidated, the dimeric state of PRC2 is likely to have major biological consequences in terms of binding and recognition of its ligands and co-factors. Variation of subunit composition within the dimeric complex is likely to affect PRC2 function as well. Thus, we propose the PRC2 dimer to be a large multisubunit complex that forms various types of interactions simultaneously with its protein and nucleic acid ligands and co-factors, allowing for its broad functional spectrum driving the initiation, propagation, maintenance and inheritance of the repressed chromatin state.

## SUPPLEMENTARY DATA

Supplementary Data are available at NAR Online.

SUPPLEMENTARY DATA
