# Supplementary material for: A dimeric state for PRC2
Source: Nucleic Acids Res. 2014 Jul 3;42(14):9236–48. doi: 10.1093/nar/gku540 (PMC4132707; doi:10.1093/nar/gku540)
Supplement: SUPPLEMENTARY DATA [file supp_42_14_9236__index.html]

A dimeric state for PRC2 — A dimeric state for PRC2 — A dimeric state for PRC2 — SUPPLEMENTARY DATA 

# A dimeric state for PRC2

## SUPPLEMENTARY DATA

**Files in this Data Supplement:**

- SUPPLEMENTARY DATA
