# Supplementary material for: A dimeric state for PRC2
Source: Nucleic Acids Res. 2014 Jul 3;42(14):9236–48. doi: 10.1093/nar/gku540 (PMC4132707; doi:10.1093/nar/gku540)
Supplement: SUPPLEMENTARY DATA [file supp_gku540_nar-03695-r-2013-File009.pdf]

Supplementary Figures

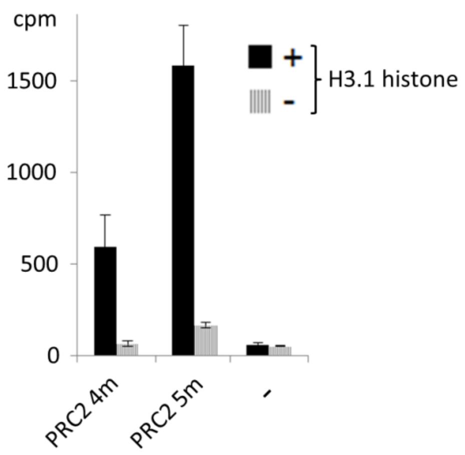

Supplementary Figure S1. Histone methyltransferase (HMTase) assay confirmed activity of recombinant human PRC2. Assay performed in the presence and absence of H3.1 histone substrate and in the presence or absence of PRC2 4m and PRC2 5m. Error bars indicating standard deviation (n = 3).

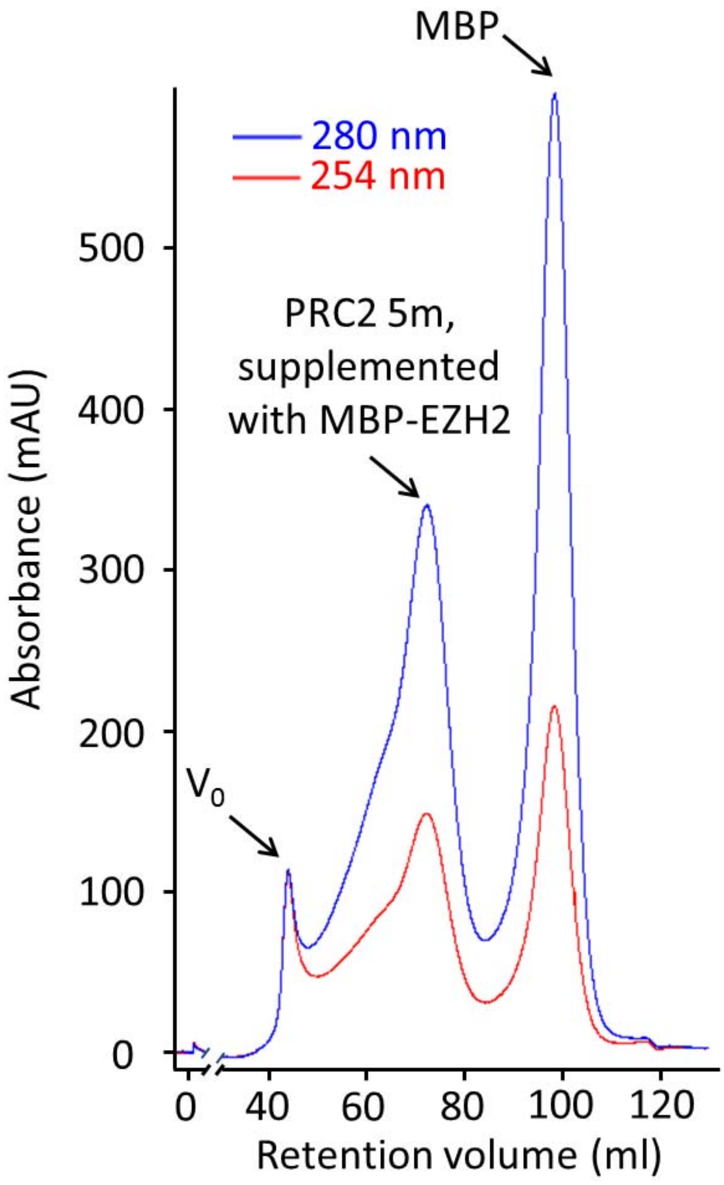

Supplementary Figure S2. FPLC-SEC purification of PRC2 5m supplemented with MBP-EZH2, in addition to an untagged EZH2. HiPrep 16/60 Sephacryl S-400 HR used for fractionation and ratio of absorbance at 280 nM to 254 nM used to confirm the absence of nucleic acid contaminants.

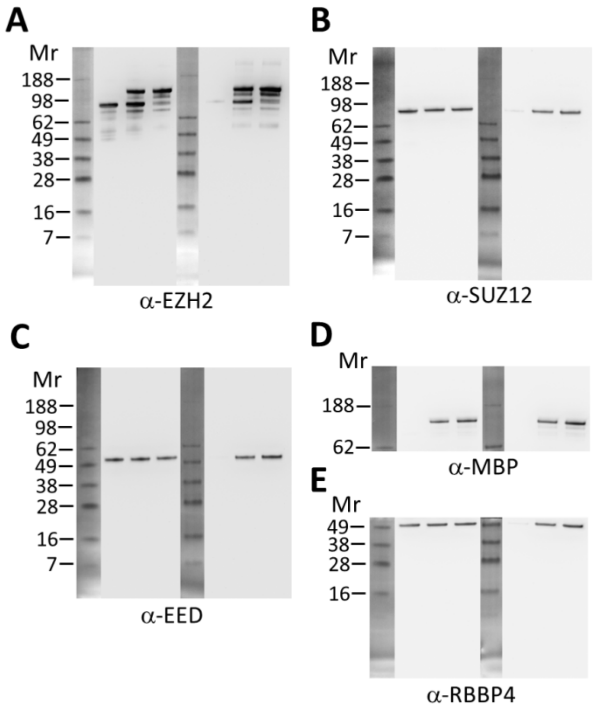

Supplementary Figure S3. Complete immunoblots used to generate Figure 4B. See Supplementary Table S1 for antibody descriptions and titers used. M_r, protein standards, masses given in kDa.

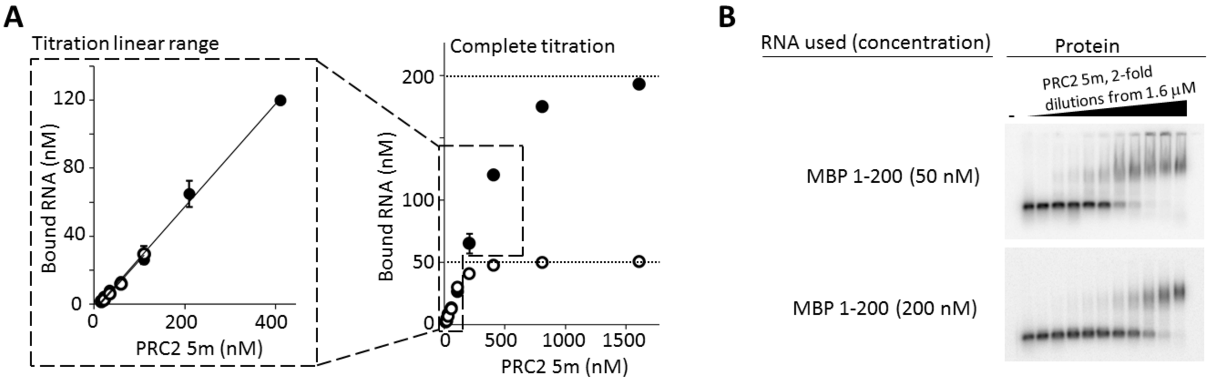

$$\text{Slope} = \frac{\text{Bound RNA (nM)}}{\text{PRC2 5m (nM)}} \rightarrow \text{Slope}^{-1} = \frac{\text{PRC2 5m (nM)}}{\text{Bound RNA (nM)}}$$

Key	RNA (concentration)	slope	slope^{-1}	$(\text{slope}^{-1})/2$	R^2
●	MBP 1-200 (50 nM)	0.298	3.4	1.7	0.991
○	MBP 1-200 (200 nM)	0.303	3.3	1.6	0.996

Supplementary Figure S4. Titration experiment to identify PRC2:RNA binding stoichiometry. (A) Titration plot indicating the concentration of bound MBP 1-200 RNA as a function of PRC2 concentration, assuming a monomer (right, complete titration range; left, titration linear range). Linear slope was used to calculate binding stoichiometry of PRC2 monomer. Under the assumption of one RNA molecule per complex, the reciprocal slope represents the number of PRC2 monomers bound to an RNA molecule. Half the reciprocal slope represents the average amount of PRC2 dimers bound to a single RNA. Experiments performed using two different RNA concentrations and repeated twice. (B) Representative EMSAs that were used to derive titration curves.

Supplementary Tables

Antigen	Primary antibody			Secondary antibody		
	Vendor	Cat #	Titer	Vendor	Cat #	Titer
EZH2	Millipore	17-662	1:10,000	Jackson ImmunoResearch	715-035-150	1:10,000
SUZ12	Santa Cruz Biotechnology, Inc.	sc-67105	1:500	Santa Cruz Biotechnology, Inc.	sc-2030	1:5,000
EED	Millipore	17-10034	1:5,000	Santa Cruz Biotechnology, Inc.	sc-2030	1:5,000
RBBP4	Abcam	ab92344	1:2,000	Santa Cruz Biotechnology, Inc.	sc-2030	1:5,000
MBP	NEB	E8038S	1:5,000	-	-	-

Supplementary Table S1. Antibodies used for immunoblotting. In case where no secondary antibody is indicated, primary antibody was conjugated to horseradish peroxidase (HRP).

HPLC peak	Peptides match to human PRC2 subunits	Fraction of peptides match to human PRC2 subunits	Number of unidentified peptides	Human PRC2 subunits that were identified
1	19	95%	1	AEBP2
2	19	95%	1	SUZ12
3	25	100%	0	SUZ12, EZH2, RBBP4
4	14	66%	7	EZH2, RBBP4, EED
total	77	90%	9	EZH2, SUZ12, EED, RBBP4, AEBP2

Supplementary Table S2. PRC2 purity and subunit identity confirmed by Mass Spectrometry. To assess the purity of the complex, 15 ug protein was fractionated over a Microbore HPLC column (0.5 x 50mm, polystyrene-divinylbenzene copolymer) and resolved into four peaks. Each peak was collected, digested with trypsin, desalted, and analyzed by flow injection with an ESI Fourier Transform Ion Cyclotron Resonance Mass Spectrometry (9.4T magnet; +/- 0.01 amu mass accuracy). 90% of the tryptic peptides were identified with high significance as being derived from human PRC2. Unidentified tryptic peptides could represent impurities, modified PRC2 peptides, or insect PRC2 peptides, but they were neither numerous nor abundant.
